# Education Research: Epilepsy Monitoring Unit Staff Education Using a High-Fidelity Manikin: A Pre-Post Intervention Study

**DOI:** 10.1212/NE9.0000000000200120

**Published:** 2024-03-13

**Authors:** John R. McLaren, Fábio A. Nascimento, Joshua Chakranarayan, Marcia Olandoski, Poornachand Veerapaneni, Jay R. Gavvala

**Affiliations:** From the Department of Neurology (J.R.M.), Boston Children's Hospital, MA; Department of Neurology (F.A.N.), Washington University School of Medicine, St. Louis, MO; Department of Adult Neurology (J.C., P.V.), Baylor College of Medicine, Houston, TX; School of Medicine (M.O.), Pontifícia Universidade Católica do Paraná, Curitiba, Brazil; and Department of Neurology (J.R.G.), McGovern Medical School, Houston, TX.

## Abstract

**Background and Objectives:**

Given the inherent risks of seizure provocation in the epilepsy monitoring unit (EMU), both miscommunication and incomplete training about the importance of when and why certain measures are taken can cause critical gaps in care for patients in an especially vulnerable state. To provide a framework that would help minimize these potential pitfalls, our objectives were 2-fold: (1) identify deficits in EMU safety and assessment using a checklist of predetermined items, including key measures essential to the care of EMU patients and (2) develop a simulation training program to address these deficits with education for staff on optimal practices.

**Methods:**

After creation of an EMU safety checklist, authors retrospectively reviewed video from 12 consecutive patients (time zero; T0) admitted to the Baylor St. Luke's EMU to assess checklist compliance and seizure response times (both electrographic and clinical). EMU staff were then trained in small teams with the help of a simulation program developed using a high-fidelity manikin. After training was complete, EMU practices and response times were reassessed in short-term (T1) and long-term (T2) follow-up intervals.

**Results:**

When all 3 groups were compared, significant behavioral improvements (Kirkpatrick level 3) were seen in several critical evaluation and safety measures. Statistically significant improvements from T0 to T1 (*p* < 0.05) were seen in orientation assessment, speech assessment, motor assessment, oxygen administration, and vital sign collection. Score improvement persisted at T2 but showed a relative decline over time in 11 of 14 measures.

**Discussion:**

Education of staff in the EMU is paramount to ensure appropriate assessment of the seizure semiology and patient safety measures. Implementation of a novel simulation-based education platform demonstrated wide-ranging improvements in staff performance of safety and testing measures. Stratification between short-term and long-term assessment periods shows that while many categories showed overall improvement, regular training may be needed to sustain improvements in assessment and patient safety. Multicenter longitudinal studies assessing the efficacy of this or similar interventions should be performed to identify best patient practices.

## Introduction

The EEG is the gold standard test for establishing a definite diagnosis of epilepsy. In cases of uncertain clinical diagnosis or evaluation of refractory epilepsy, an inpatient EEG admission in an epilepsy monitoring unit (EMU) is required to obtain simultaneous recording of EEG data and video to capture stereotyped patient events. Measures to induce seizures are typically used such as weaning antiseizure medication(s) and provocative maneuvers including sleep deprivation, photic stimulation, and hyperventilation.^1^

The act of provoking seizures, however, poses inherent risks to the patient, most notably the potential for injuries and cardiopulmonary complications, including sudden unexpected death in epilepsy.^2-5^ Furthermore, in patients undergoing a presurgical evaluation, it is insufficient to simply document stereotyped patient events because there is significant value in the staff's ability to characterize salient features of clinical semiology, including a detailed description of auras, the presence or absence of awareness, and the progression of ictal and postictal signs and symptoms.^6^

These intricacies highlight the challenges of optimal functioning in an EMU. A successful unit must balance appropriate testing for the purposes of characterizing events with the paramount goal of ensuring patient safety. For this to be achieved, the ideal unit would maintain appropriate communication and safety protocols with continuing education for all staff members involved in patient care. However, there are no recognized guidelines for training personnel or best practices for maximizing patient safety.^7^ Some of this has been previously addressed through implementation of consensus recommendations at the level of individual institutions^8,9^ and larger regional and national workgroups.^10^ However, focus on continuing education for staff has not been adequately emphasized in the literature.

To address this gap in clinical care, we aimed to assess the short-term and long-term effectiveness of a simulation-based training platform for staff in the EMU using a pre-post interventional study design. Our first objective was to characterize the baseline practice of safety and testing assessments in the Baylor Comprehensive Epilepsy Center, using a scoring checklist of critical seizure evaluation and safety measures. Subsequently, we implemented a novel simulation-based training program for all EMU staff with a high-fidelity manikin. After training was complete, we reassessed safety and testing assessments over the subsequent 6 months, divided into short-term and long-term intervals.

## Methods

The Baylor St. Luke's EMU is an open unit on the neurosciences floor composed of nurses and patient care technologists who rotate care among all patients on the floor, in addition to EEG technologists who provide continuous EEG monitoring of the admitted EMU patients. A pre-post interventional study design was chosen to assess the effect of the simulation program on the quality of safety and testing assessments. While a control group would add more scientific rigor, the practicalities of implementing a control group in an open nursing unit, including the potential deleterious impact to patient safety and care, would have made the study unfeasible. This study was conducted in accordance with the STROBE checklist.

### Baseline Video Review

Authors J.C. and J.R.G. developed a checklist to assess the performance of established EMU safety and monitoring practices based on previously published consensus recommendations^8,9^ (eTable 1, links.lww.com/NE9/A59). In the preintervention phase, the same authors retrospectively reviewed video EEG (vEEG) data of consecutive patients admitted to Baylor St. Luke's EMU from July 2021 to September 2021 whose stereotyped spells were captured and determined to be epileptic per vEEG data. Patients met inclusion criteria if they had electrographically confirmed (epileptic) stereotyped events captured with clinical motor features. Patients with purely electrographic seizures or focal aware seizures with nonmotor onset without EEG correlate were excluded. Data extracted from video review included staff member compliance with checklist items, time of electrographic seizure onset, time of clinical seizure onset, and time of staff first response to the events. The staff response was determined by video review and included either the EEG technologist intercom response or nursing staff entrance into the patient room. Patient demographics are summarized in Table 1.

**Table 1:** Demographics of Patients Included in the Video Analysis of Staff

	Preintervention (T0)	Short-term postintervention (T1)	Long-term postintervention (T2)	*p* Value
No. of patients (total no. of seizures)	12 (69)	17 (47)	15 (55)	
Age, mean ± SD	32.6 ± 10.1	43.4 ± 17.0	40.3 ± 11.4	0.112
Gender (female %)	50	58.8	60	0.853
Seizure type (focal %)	83.3	82.3	80	0.973

Demographics for each comparator group including T0 (preintervention before simulation training), T1 (short-term post intervention, 1–3 months after training) and T2 (long-term post intervention, 3–6 months after training) are summarized in the table. Statistical comparisons between the groups were performed using 1-way ANOVA for age and the χ^2^ test for gender and seizure type with *p* < 0.05 representing statistical significance.

### Simulation Training Program

All staff on the Baylor St. Luke's Neuroscience floor were expected to complete the simulation laboratory exercise and debriefing as part of their annual competency measures. Staff were notified 1 month in advance of the training exercise and were provided a total of 8 training session times; in all cases, staff were allotted time during their normal work hours to avoid taking overtime. In advance of the session, staff were given verbal objectives at weekly staff huddles and provided a PowerPoint highlighting EMU safety expectations. These expectations were the same ones that helped inform the aforementioned behavioral assessment checklist.

**Figure 1:**
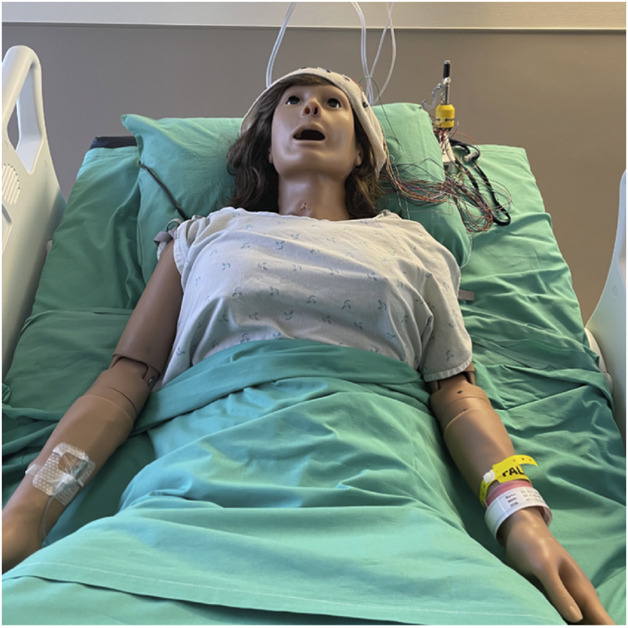
High-Fidelity Manikin Used During Simulation Case

A simulation training program was developed using a high-fidelity manikin with a customized simulation session that mimicked a real-life EMU scenario (Table 2). The Baylor St. Luke's Simulation laboratory has a Gaumard Susie High-Fidelity Simulation Manikin (Figure 1) that was used for the purposes of this study. Each training session was allotted 1 hour of time in the Baylor St. Luke's simulation laboratory that included 5 minutes for an initial briefing of the laboratory setup and expectations, 15 minutes for the simulation scenario, and 40 minutes for debriefing and discussion about optimal expectations.

**Table 2:** Simulation Case Description, Learning Objectives, and Critical Actions

Brief narrative description of case	Patient is a 24-year-old woman with history of febrile seizures and drug-resistant epilepsy admitted to the EMU for seizure classification as part of presurgical workup for epilepsy surgery
Primary learning objectives	Learners should be able to: 1. Demonstrate ability to perform comprehensive assessment of a patient having a seizure in the EMU 2. Recognize patient safety issues during a seizure and apply appropriate measures to always ensure patient safety
Critical actions	1. Upon entering the room, ensure camera view is unobstructed, ensure there is adequate lighting in the room for the camera, and uncover patient 2. The learner immediately asks the patient what they are feeling, encouraging the patient to speak up in an audible voice and repeating what the patient is saying in a loud voice. Ask the patient if this is their typical aura. They then proceed to ask orientation questions asking the patient for their name, where they are, and the date 3. Ask the patient “repeat after me and remember the following phrase, I will ask you about it later”: red cow or blue monkey, etc. 4. Ask the patient to follow a command without showing them: please hold up 3 fingers then ask the patient to repeat on the other side 5. Ask the patient whether they remember the phase given earlier 6. As seizure progresses into loss of awareness, the abovementioned assessment is repeated at the same time describing any signs the patient is exhibiting in a loud voice: mouth or hand movements, facial expression, etc 7. As the seizure progresses into head version, learner immediately realizes GTC seizure is imminent and institutes safety measures: turn patient on their side, monitor vital signs, perform suctioning and O_2_ administration and stand by to give ASM per protocol 8. As patient enters postictal phase, continue vital signs monitoring and patient assessment until patient returns to baseline

Abbreviations: ASM = antiseizure medication; EMU = epilepsy monitoring unit; GTC = generalized tonic-clonic.

The simulation session began with the “patient” (Table 2) pushing the button for a habitual aura (epigastric “rising” sensation). It was expected that the staff would enter the room and assess and interact with the patient. In approximately 1 minute, the manikin would progress to a focal impaired awareness seizure with oral and hand automatisms. It was expected that staff would recognize the behavioral arrest and change in semiology. After an additional 30 seconds, the manikin had progression of the ictal event to a bilateral tonic-clonic seizure. In the postictal period, the “patient” gradually regained awareness with appropriate responses to orientation questions and the ability to follow commands but had unilateral weakness (Todd's paresis) before progressive return to full strength. General examination findings included progressive tachycardia, tachypnea, and hypoxemia. Staff who participated in the simulation were expected to interact with the manikin, recognize changes in clinical state, and perform appropriate safety measures. Training was completed in teams of 3–4 staff members, typically composed of a combination of nursing staff, patient care technologists, and 1 EEG technologist. Members of the group were instructed to take a “divide and conquer” approach, each performing different tasks and assuming different roles that they determined on their own. Immediately following the simulation exercise, a debriefing session occurred led by author J.R.G., the nursing manager of the neuroscience floor, and the neuroscience educator, all of whom were present in the simulation room. In the debriefing, these team leaders identified strengths and weaknesses of the simulation laboratory exercise and reinforced optimal practices by reviewing best practice slides that were previously provided to staff.

### Interval Follow-Up Assessments

Two retrospective reassessments of EMU safety and evaluation practices from the initial checklist were performed after all staff had completed the simulation training program in September 2021.

### Statistical Analysis

IBM SPSS Statistics version 28 was the primary tool used to assess statistical significance of collected data. A Kruskal-Wallis test was first used as a nonparametric method to compare the 3 independent (abnormally distributed) groups of median time points for clinical and EEG response. The Dunn test with the Bonferroni correction was used to further compare each possible combination of 2 groups. A χ^2^ test with the Bonferroni adjustment was performed to compare the 3 treatment groups (preintervention, short-term post intervention, and long-term post intervention) and assess significant differences in compliance. The Fisher exact test with the Bonferroni adjustment was used to further compare each possible combination of 2 groups, once again assessing for any significant differences in compliance. Any episode in which a compliance item was not indicated was considered missing data for analysis. While initial data collection differentiated between “yes,” “no,” or “partial” compliance, any partial response was considered a “no” during analysis.

### Standard Protocol Approvals, Registrations, and Patient Consents

This study was approved by the Baylor College of Medicine and Affiliated Hospitals Institutional Review Board (approval no. H-48272).

### Data Availability

On reasonable request, the data that support the findings of this study are available from the corresponding author (J.R.G.).

## Results

In total, 54 staff participated in the simulation training sessions (8 EEG techs, 26 nurses, and 20 patient care technologists). For the baseline video review, a total of 12 patients with 69 epileptic seizures met inclusion criteria between July and September 2021 and were reviewed by staff for the preintervention analysis. Using the same preassessment inclusion and exclusion criteria, the short-term reassessment occurred in April 2022 for EMU admissions from October to December 2021 (47 episodes, 17 patients). The long-term reassessment also occurred in April 2022 for EMU admissions from January to March 2022 (55 episodes, 17 patients).

### Baseline Video Review (T0)

**Figure 2:**
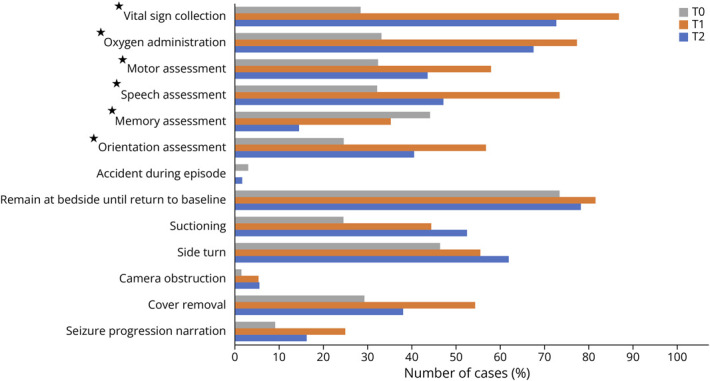
Evaluation and Safety Checklist Item Compliance Between Groups Percentage of cases demonstrating compliance with evaluation and safety checklist during preintervention period before stimulation training (T0), short-term post intervention, 1–3 months after training (T1), and long-term post intervention, 3–6 months after training (T2). Statistical analysis performed with the χ^2^ test, comparing T0 vs T1 vs T2. Starred items indicate statistical significance, *p* < 0.05.

Clinical features of patients included in the baseline video review are summarized in Table 1. Initial video review revealed a median staff response time from electrographic onset of 33 seconds (Q1–Q3: 18–64 seconds) and a median staff response time from clinical onset of 22 seconds (Q1–Q3: 8–49 seconds) (Table 3). There were low levels of compliance with safety and evaluation checklist measures (Figure 2) because only 1 item was completed greater than 50% of the time. Particularly low compliance items included subjective experience assessment (8.1%), seizure progression narration (9.1%), orientation assessment (24.6%), suctioning (24.5%), vital sign collection (28.4%), cover removal (29.3%), and oxygen administration (33.3%). More positively, accidents only occurred in 3% of cases, and the camera was blocked in only 1.5% of cases. While there were often attempts at completing checklist items, many were not done according to protocol. Together, these results suggested that both awareness of protocol and proper technique were areas of concern.

**Table 3:** Response Time From Electrographic and Clinical Seizure Onset Between Groups

	T0 (median; Q1–Q3)	T1 (median; Q1–Q3)	T2 (median; Q1–Q3)	*p* Value^a^
Time EEG	33.00 (18.00–63.00)	30.00 (19.00–57.50)	29.50 (19.25–46.00)	0.802
Time clinical	22.00 (8.00–49.00)	37.50 (25.00–60.00)	34.00 (16.75–54.75)	**0.004**

	Subgroup analysis		
	*p* Value^b^ (T0 × T1)	*p* Value^b^ (T0 × T2)	*p* Value^b^ (T1 × T2)
Time clinical	**0.003**	0.181	0.456

T0: preintervention before simulation training; T1: short-term post intervention, 1–3 months after training; T2: long-term post intervention, 3–6 months after training.

Bold indicates statistical significance, *p* < 0.05.

a Kruskal-Wallis test.

b Dunn test with Bonferroni adjustment.

### Early Postintervention (T1)

Clinical features for the early and the late reassessment periods are summarized in Table 1. In the early reassessment period, significant improvements were seen broadly across both the safety and evaluation subcategories, with 8 checklist items completed greater than 50% of the time. These included cover removal (54.3%), side turn (55.6%), orientation assessment (56.8%), motor assessment (57.9%), speech assessment (73.5%), oxygen administration (77.3%), remaining at bedside until return to baseline (81.6%), and vital sign collection (86.8%) (Figure 2). Of importance, there were no relative declines for any checklist items and no recorded accidents. Compared with the preassessment baseline, time to response from EEG onset was slightly but not significantly faster (T0 median: 33.0 seconds; T1 median: 30.0 seconds; *p* > 0.05); however, the response time from clinical onset was also longer and statistically significant (T0 median: 22.0 seconds; T1 median: 37.50 seconds; *p* = 0.003). Overall, compliance with checklist items had largely been improved, while there was some variability with staff response time.

### Late Postintervention (T2)

In the long-term reassessment period, there were relative improvements from baseline in 12 of 14 checklist items, with only relative decline in memory assessment (14.5%) and camera obstruction (5.6%); however, improvements were not as distinct as they were in the early postintervention group because there were relative declines from the early postintervention data in 11 of 14 measures (Figure 2). Reassuringly, accidents remained low (1.8%). There were no statistically significant differences in the late postintervention group's EEG or clinical response time from either the baseline assessment group or the early postintervention group (*p* > 0.181, all groups).

### Between-Group Comparison

Between-group comparisons of baseline with the short-term and long-term interval reassessments revealed statistically significant improvements in 3 of 8 evaluation checklist items (orientation assessment, *p* = 0.005; speech assessment, *p* = 0.001; and motor assessment, *p* = 0.041) and in 2 of 5 safety measures (oxygen administration and vital sign collection, both *p* < 0.001) (Figure 2). One task was significant for declining performance (memory assessment, *p* = 0.002). In subgroup analysis of those checklist items that reached statistical significance in between-group analysis, there were statistically significant improvements from baseline to the early postintervention group (T0 × T1) in orientation assessment (*p* = 0.006), assessment of the patient's ability to speak (*p* < 0.001), motor task assessment (*p* = 0.042), oxygen administration (*p* < 0.001), and vital sign collection (*p* < 0.001). Meanwhile, there were statistically significant improvements from baseline to the late postintervention group (T0 × T2) in only 2 measures, including oxygen administration (*p* = 0.006) and vital sign collection (*p* < 0.001), with 1 task demonstrating significant decline in performance (memory assessment, *p* < 0.001). There were no statistically significant differences between early and late postassessment groups (T1 × T2) (Table 4).

**Table 4:** Evaluation and Safety Checklist Item Compliance Subgroup Analysis

	*p* Value^a^ (T0 × T1)	*p* Value^a^ (T0 × T2)	*p* Value^a^ (T1 × T2)
Orientation assessment	**0.006**	0.228	0.417
Memory assessment	1	**<0.001**	0.105
Speech assessment	**<0.001**	0.372	0.078
Motor assessment	**0.042**	0.756	0.627
Oxygen administration	**<0.001**	**0.006**	1
Vital sign collection	**<0.001**	**<0.001**	0.384

T0: preintervention before simulation training; T1: short-term post intervention, 1–3 months after training; T2: long-term post intervention, 3–6 months after training.

Bold indicates statistical significance, *p* < 0.05.

a Fisher exact test with Bonferroni adjustment.

## Discussion

Safety and evaluation training for all staff in the EMU is of paramount importance in the optimal assessment and care of patients in the EMU. Patients in the EMU are prone to development of status epilepticus, seizure-related falls or injuries, psychiatric complications, and cardiac/respiratory complications, with pooled analyses suggesting serious adverse events occurring in up to 10% of patients.^3,9^ Furthermore, objective characterization of the ictal semiology has proven valuable in the characterization of the epileptic zone for surgical evaluations.^11-13^

The use of checklists and consensus guidelines have been used in the literature as potential methods to assess and improve EMU care. However, the use of these tools in conjunction with simulation-based training have been sparsely reported, with mixed results, for sustained behavioral modification.

In our initial baseline assessment, we found opportunities for improvement in patient care for both “evaluation” checklist items, which help to further characterize seizures, and “safety” items, which more directly protect patients through complication prevention and mitigation. Following development and implementation of a simulation laboratory training session to all staff in the EMU, there were statistically significant improvements in orientation assessment, speech assessment, motor assessment, oxygen administration, and vital sign collection in the short-term reassessment. In the long-term reassessment, there were many trends toward improvement, but statistically significant improvements were seen only in oxygen administration and vital sign collection (Kirkpatrick level 3)^14^ with a statistically significant decline in memory assessment. No significant changes were noted in time of responsiveness by staff in the EMU.

While historically, skill-based assessments have been centered around reading of descriptive text and/or observation of a procedure with the expectation to complete the task thereafter, recent studies in medical education outside the EMU have illustrated that the use of simulation-based training is an effective strategy to ensure standard of care is met across all staff.^15^ These simulations allow for individuals to hone their skills in a risk-free environment and promote development of critical team-based skills, including communication, group decision-making, coordination of safety, and testing checklist items.^16^ For training to be effective, it must accurately model the essential components of epilepsy safety and facilitate transfer of these skills to the real-world setting.^17^ Our training model aimed to do so by emphasizing a realistic clinical scenario along with a standard treatment team to emphasize coordination of the required safety and evaluation measures.

We are aware of only 1 study that has used interprofessional simulation as a model for EMU training; however, this did not lead to a significant performance improvement.^18^ Two other groups developed standardized, nonsimulation-based ictal examination education, one of which did not lead to sustained improvements in the postintervention period^19^ and the other showing reduction in missed seizures but not for falls.^20^

Currently, there are no available recognized guidelines for training personnel or best practices for maximizing patient safety in the EMU; we believe that the baseline knowledge and practice reflected this. Furthermore, we believe the current nursing model of an open EMU unit additionally diluted staff familiarity and comfort in best care practices in an EMU setting. Before the development of this educational vignette, staff education was focused on general neuroscience care and included 1 dedicated lecture on seizures. In our opinion, this is much too brief to encompass the nuances of optimal EMU care. Due to emphasis of patient safety in nursing education, there was inherently more compliance in performing safety measures than testing. However, with more dedicated education, there was a significant corresponding improvement, highlighting the importance for seizure-specific safety and testing protocols for all staff in the EMU.

The most robust changes from preintervention to postintervention assessment were in oxygen administration and assessing vital signs. It was unclear why these functions changed the most because there was not a greater emphasis placed on these items during the training period. One possible explanation could be the open EMU layout of the neuroscience floor, where staff are focused on responding to acute neurologic emergencies. Naturally, this response typically involves an assessment of vital signs and oxygen administration. In future iterations, this may change the focus of the educational sessions to more heavily weight other safety items that were less impressive. Of note, there was a considerable drop-off in compliance from short-term to long-term follow-up, including a statistically significant decline in memory assessment. The reason for this decline can potentially be explained by a variety of factors. Decay of training emphasis over time was a likely contributor to the decline because enthusiasm and emphasis on training principles are often highest in the period directly after training.^21^ In addition, a possible contributing factor could have been the development of “shortcuts” in testing and safety over time. Given the necessity to perform checklist measures for every episode, the development of heuristics, which may not directly fulfill checklist items, may have been evident. Finally, it is possible that turnover of EMU staff, with several staff members hired after the training intervention, could have contributed to lower scores over time. It is unclear why memory assessment would decline from baseline testing, but the EMU setting may offer some insight. Assessments of orientation, speech, and motor function are standard parts of a neurologic examination in a mixed neurosciences unit, whereas memory assessment is fairly specific to the care of a patient with seizures. Thus, with appropriate initial instruction, staff are able to fall back on their familiarity with the neurologic examination, but it is harder to educate staff on the nuances of acute seizure care without reinforcement. This decline highlights the need for additional effort in addressing this aspect of patient assessment. Regardless, the decline in compliance over time suggests the need for regularly scheduled training as a measure to maintain improved compliance. This would allow for continual adjustment in the case details, with specific emphasis placed on checklist items exhibiting the largest decreases in compliance. Interval education would also allow for more experienced staff to both review basic protocol and provide insight to newer staff on the ways in which training may or may not match up with real-world scenarios.

In addition to checklist compliance, the response time to an episode was also assessed during retrospective review. When the 3 groups were compared, no significant improvements were seen in response time from electrographic onset. However, there was a statistically significant increased latency in response time from clinical onset between all groups, with subgroup analysis showing a significant difference only between the baseline and short-term follow-up. This result illustrates variability in this metric and is not entirely unexpected given that training intervention focused on timely completion of checklist items and not on improving initial response time or detection of electrographic seizures. Dedicated interventions designed to increase early detection of clinical and electrographic signatures of a seizure are likely needed to significantly improve response time.

In another center's assessment of their own response time,^22^ hospital care team members, like those in our EMU, responded on average 22 seconds from the clinical event, the same as our baseline; however, they did not separately assess response from electrographic onset. They also assess caregiver response time, which was, on average, twice as fast, and would be worth investigating in future iterations.

There are several limitations to our study, the clearest of which was that it was conducted at a single EMU, with only a 6-month follow-up observation period. This, understandably, led to a smaller sample size of staff members and retrospective cases to review. Other reports have clearly documented significant variability between institutions, with cultural factors that differ geographically, including size, standard practices, nurse-to-patient ratios, nursing skill level, specialty nursing support, and EEG technician availability.^23-25^ While this raises legitimate questions about the generalizability of our result, the methodology and approach described earlier should be of use to many other EMUs in the absence of standardized safe care practices that are applicable to the heterogenous EMUs around the country. Ultimately, individualized staff education and nursing best practice recommendations need to be developed with baseline EMU nursing competencies, skills, and knowledge in mind. Furthermore, only 1 simulation vignette was used for the purposes of training and on 1 high-fidelity manikin. While this case encompassed salient findings of care for a patient with epilepsy, including cases with other semiologic features of an epileptic seizure, nonepileptic events, other patient age groups, and the use of different manikin types, including more low-fidelity models, may further enhance the generalizability of these findings. Finally, the lack of a comparative group is a notable weakness, and subsequent studies should be performed to assess the impact of such an education model when compared with traditional didactic sessions.

Future directions for investigations of this kind should involve expansion to other EMUs and the implementation of long-term follow-up in the context of regularly scheduled training. Dedicated training should also focus on improving recognition of electrical and clinical seizure onsets to improve the timeliness of safety and testing assessments. Furthermore, the utility of such an intervention may exist beyond the confines of an EMU. Such simulation models may be of value for other trainees and staff including medical students, nurses, and residents who may be called to assess a patient having an epileptic seizure or seizure-like activity in the emergency department or other areas of the hospital. Because there are no current evidence-based guidelines for EMU safety and education from any of the major domestic or international epilepsy societies, it is our hope that this study will help illustrate that it is possible to have a successful behavior-modifying intervention in EMU with modern educational techniques and that there is a need for continued advocacy for EMU safety and evaluation guidelines to ensure minimum standards are met.

## Appendix Authors

**Table:**

Name	Location	Contribution
John R. McLaren, MD	Department of Neurology, Massachusetts General Hospital, Boston	Drafting/revision of the article for content, including medical writing for content; analysis or interpretation of data
Fábio A. Nascimento	Department of Neurology, Washington University School of Medicine, St. Louis, MO	Drafting/revision of the article for content, including medical writing for content; analysis or interpretation of data
Joshua Chakranarayan, MD	Department of Adult Neurology, Baylor College of Medicine, Houston, TX	Major role in the acquisition of data; study concept or design
Marcia Olandoski, MD, PhD	School of Medicine, Pontifícia Universidade Católica do Paraná, Curitiba, Brazil	Analysis or interpretation of data
Poornachand Veerapaneni, MD	Department of Adult Neurology, Baylor College of Medicine, Houston, TX	Major role in the acquisition of data
Jay R. Gavvala, MD, MSCI	Department of Neurology, McGovern Medical School, Houston, TX	Drafting/revision of the article for content, including medical writing for content; major role in the acquisition of data; study concept or design; and analysis or interpretation of data

## Glossary

EMU: epilepsy monitoring unit
vEEG: video EEG
